# Diagnostic potential of endothelin-1 in peri-implant diseases: a cross-sectional study

**DOI:** 10.1186/s40729-024-00551-0

**Published:** 2024-06-14

**Authors:** Yoshiki Saito, Tomotaka Nodai, Takashi Munemasa, Taro Mukaibo, Yusuke Kondo, Chihiro Masaki, Ryuji Hosokawa

**Affiliations:** Division of Oral Reconstruction and Rehabilitation, Kyushu Dental University, Kitakyushu, Fukuoka 803-8580 Japan

**Keywords:** Endothelin-1, Peri-implant mucositis, Peri-implantitis, Interleukin-1β, Marginal bone loss

## Abstract

**Purpose:**

This study aimed to evaluate the potential of Endothelin-1 (ET-1), a peptide derived from vascular endothelial cells, as a biomarker for diagnosing peri-implant diseases.

**Methods:**

A cohort of 29 patients with a total of 76 implants was included in this study and subsequently divided into three groups based on peri-implant clinical parameters and radiographic examination: healthy (peri-implant health) (*n* = 29), mucositis (*n* = 22), and peri-implantitis (*n* = 25) groups. The levels of ET-1 (ρg/site) and interleukin (IL)-1β (ρg/site) in peri-implant sulcus fluid (PISF) samples were determined using enzyme immunoassay. Statistical analyses were conducted using Kruskal–Wallis and Steel–Dwass tests. Logistic regression and receiver operating characteristic (ROC) curve analyses were performed to evaluate the diagnostic performance of the biomarkers.

**Results:**

ET-1 levels were significantly elevated in the peri-implantitis group compared to those in the healthy group, and were highest in the peri-implant mucositis group. Additionally, IL-1β levels were significantly higher in the peri-implantitis group than those in the healthy group. ROC curve analysis indicated that ET-1 exhibited superior area under the curve values, sensitivity, and specificity compared to those of IL-1β.

**Conclusions:**

Our findings suggest that the presence of ET-1 in PISF plays a role in peri-implant diseases. Its significantly increased expression in peri-implant mucositis indicates its potential for enabling earlier and more accurate assessments of peri-implant inflammation when combined with conventional examination methods.

## Background

The number of patients opting for dental implants as a form of prosthetic treatment has increased in recent years; however, the emergence of peri-implant diseases has raised concerns. These diseases can be classified into two types: peri-implant mucositis and peri-implantitis, with their prevalence rates ranging from 19 to 65% and 1 to 47%, respectively [1]. Peri-implant mucositis is a plaque-induced disease and is defined as a reversible soft tissue inflammatory lesion around implants without any loss of supporting bone or continuous marginal bone loss [2]. It is considered a pathological precursor to peri-implantitis, although the transition to peri-implantitis is unclear, and the clinical diagnosis is complex [3, 4]. Treatment for peri-implant diseases typically involves a combination of nonsurgical, surgical, and pharmacological therapies. However, despite Heitz-Mayfield et al. [5] reporting a cure rate of 42% at 5 years and many related procedures existing, there is no consensus on which procedure is the most effective. Given these considerations, an accurate diagnosis of peri-implant mucositis is crucial to reduce the risk of developing peri-implantitis [6].

The 2017 World Workshop proposed a classification system for peri-implant diseases and conditions, with diagnosis primarily relying on various clinical measurements such as pocket probing depths (PPD), bleeding on probing (BOP), and assessment of radiographic images [7, 8]. However, these metrics alone cannot sufficiently determine peri-implant disease activity, future crestal bone loss, or future implant failure [9]. For instance, BOP is determined based on the dichotomous presence or absence of a single parameter. To avoid excessive probing depths, it is important to apply low probing pressure (approximately 0.25 N) due to the delicate nature of peri-implant mucosal attachment [9, 10]. Moreover, a large over-contoured implant superstructure can cause traumatic BOP due to limited probing directions, leading to bleeding [9]. Therefore, a non-invasive diagnostic approach capable of accurately determining the peri-implant status is required.

Biomarker-based diagnostic strategies have emerged as effective non-invasive techniques for early peri-implant diseases detection [11]. Identifying biomarkers that are associated with peri-implant tissue destruction enables preemptive detection before clinical symptoms appear. Thus, combining these with conventional protocols can improve the accuracy of early diagnosis and prediction of disease progression. Faot et al. [12] and Ghassib et al. [13] identified biomarkers such as Interleukin 1β (IL-1β), Tumor Necrosis Factor-alpha (TNF-α), and Matrix metalloproteinase 8 (MMP-8) in the peri-implant sulcus fluid (PISF) as valuable for diagnosing peri-implantitis [14, 15]. However, early biomarker detection of peri-implant diseases remains challenging due to the lack of evidence of biomarkers with elevated levels in peri-implant mucositis; thus, we focused on peptides with established regulatory effects on inflammatory cytokines. Peptides are highly vascular-permeable due to their smaller molecular weight compared with those of inflammatory cytokines [16]. Consequently, peptides leaking from adjacent tissues due to inflammation-induced vasodilatation hold promise for the early detection of peri-implant diseases.

Endothelin-1 (ET-1), first identified by Yanagisawa et al. in 1988 [17], is a 21 amino acid peptide secreted by vascular endothelial cells with multifunctional regulatory properties. It has a small molecular weight (approximately 2.5 kDa) and acts as a potent vasoconstrictor, influencing various physiological processes and potentially impacting the progression of hypertension and inflammatory diseases [18]. Fujioka et al. [19] have shown that ET-1 levels in the gingival sulcus exudate of patients with periodontitis are significantly higher than those in the healthy gingival sulcus exudate. Rikimaru et al. [20] reported that ET-1 regulates IL-1β expression in gingival tissues. ET-1 is elevated in periodontitis and is associated with inflammatory cytokines and other factors, although its detailed effects are not clearly established [21]. Although periodontitis and peri-implantitis have different supporting tissue structures, there share many clinical features and biomarkers are consistent. However, the role of ET-1 has not been evaluated in peri-implant mucositis and peri-implantitis.

Therefore, this study aimed to investigate the association between ET-1 levels and peri-implant diseases and to determine whether ET-1 can be used as a predictive marker for peri-implant mucositis and peri-implantitis.

## Methods

### Study design

This cross-sectional study was conducted in accordance with the Declaration of Helsinki on Human Studies. All experimental protocols involving patients and healthy individual samples were approved by the Ethics Committee of Kyushu Dental University (No. 18–32). Data collection was conducted according to the Strengthening the Reporting of Observational Studies in Epidemiology guidelines.

We enrolled a cohort of 62 patients with a total of 137 implants who received maintenance care at Kyushu Dental University Hospital between July 2022 and November 2023. The inclusion criteria were: (1) age ≥ 20 years, (2) absence of pregnancy or lactation, (3) use of functioning implants for at least 12 months, (4) no history of poorly controlled systemic diseases, (5) no history of nonsurgical or surgical treatment, such as scaling at the site, to be examined within 3 months of examination, and (6) no history of medical treatment during the last 3 months before examination and sampling.

The exclusion criteria were: (1) presence of implants without previous radiographs (base data), (2) presence of implants placed in a position or with superstructures that made probing difficult, (3) presence of implants with an average marginal bone loss of ≥ 0.2 mm and BOP (-), and (4) presence of implants for which the amount of bone resorption could not be measured due to unclear radiographic images.

Ultimately, 29 patients (15 males and 14 females; mean age: 74.6 years) with a total of 76 implants were included for final analyses. All participants provided informed consent.

### Clinical evaluations

**Fig. 1:**
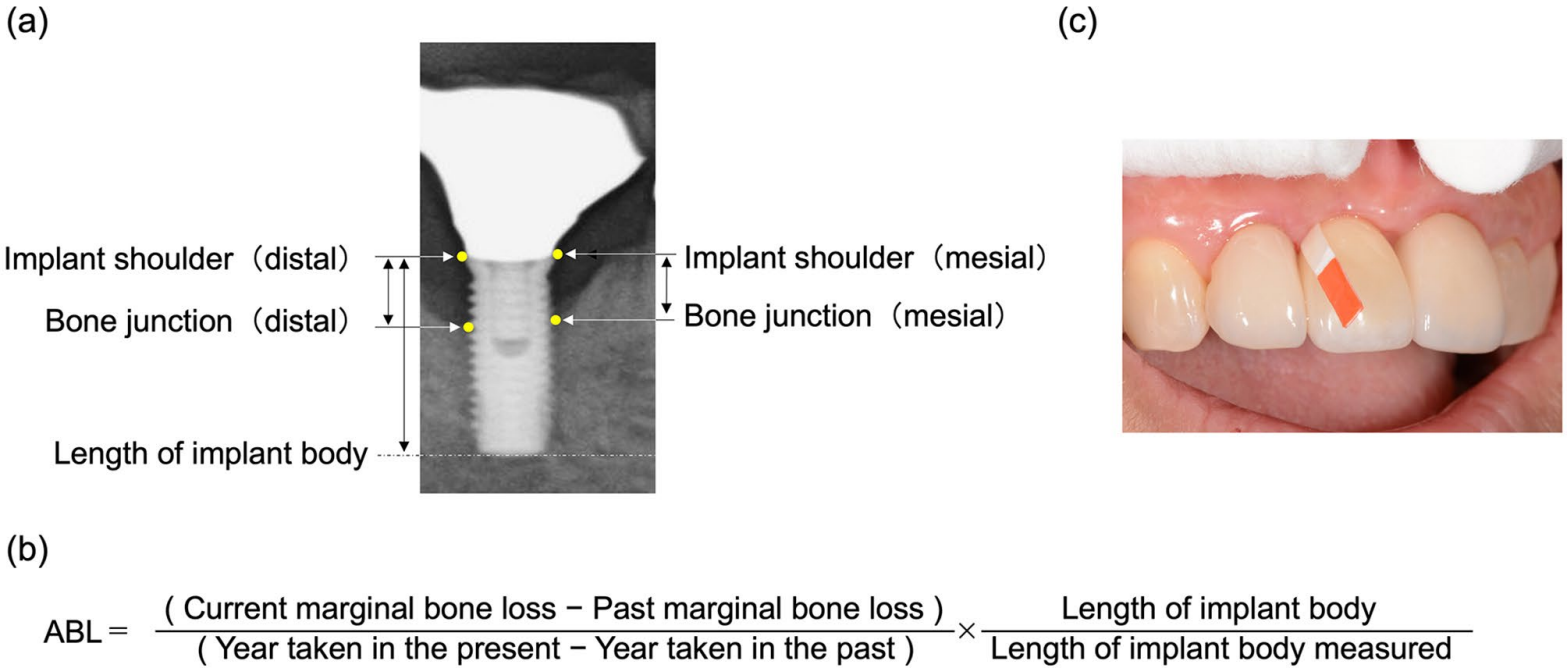
(**a**) Bone resorption measurement using radiographic images. The distance between the proximal bone junction and the most apical side of the implant was measured using the implant shoulder as a reference point. The resulting values were corrected by the magnification ratio to the long diameter of the implant body, and marginal bone loss (MBL) was calculated. (**b**) Formula for calculating average annual bone loss (ABL). The ABL around the implants was calculated in comparison to the MBL at the baseline. (**c**) The PISF was obtained using PerioPaper from the site where the PPD was deepest

The participants’ implants were assessed by a trained dentist (YS) using the following measurements: Peri-implant conditions were evaluated using a plastic probe (Colorvue, Hu-Friedy, Chicago, IL, USA) under low pressure (0.25 N) for PPD, presence of BOP, modified plaque index (mPI), and modified gingival index (mGI) [22]. Radiographic assessment involved random assignment of radiographs to each evaluator (TN, TM, and TM), with evaluators blinded to any patient-identifiable information. Radiographic examination was conducted using distance measurement software (VHX-5000, Keyence, Tokyo, Japan) in an electron microscope to measure the distance between the proximal bone junction of the implant and the most apical side of the implant, with the implant shoulder serving as the reference point. Subsequently, we calculated the average of the measurements taken by the three evaluators. Marginal bone loss (MBL) [23], was calculated by adjusting values for the magnification ratio of the length of the implant body. The average annual bone loss (ABL) around the implants was then calculated and compared with the MBL at the baseline (Fig. 1a and b).

### Patient groups

Following the 2017 World Workshop [7] guidelines, the 76 eligible implants were categorized into three groups: healthy (*n* = 29), mucositis (*n* = 22), and peri-implantitis (*n* = 25), defined by the characteristics presented below. Pathological bone resorption was defined as an ABL of ≥ 0.2 mm at the peri-implant area, according to the Toronto Conference [24].

1. Healthy group (peri-implant health): Implants with BOP (-), ABL < 0.2 mm, and no other signs of inflammatory lesions on the oral mucosa.
2. Peri-implant mucositis group (peri-implant mucositis): Implants with BOP (+) and ABL < 0.2 mm.
3. Peri-implantitis group: Implants with BOP (+) and ABL > 0.2 mm.

### PISF sampling

Plastic curettes (Implacare; Hu-Friedy, Chicago, IL, USA) were used to remove plaque above the peri-implant margin. Sampling sites were isolated using cotton rolls and dried using a gentle stream of air. PerioPaper® (OraFlow Inc.; Plainview, NY, USA) was gently inserted < 1–2 mm into the deepest sulcus until a slight resistance was felt, and then held in place for 1 min (Fig. 1c). Samples were collected five times from the same site using the same method, with a 1-minute interval between each collection. Any PerioPaper contaminated with blood or saliva was discarded and replaced after 10 min. To minimize evaporation, volume quantification was performed immediately after sampling using a Periotron 8000 device (OraFlow Inc.). The Periotron 8000 was calibrated prior to the study and recalibrated periodically, following the manufacturer’s instructions. Periotron values are expressed as the volume of PISF (µL) with reference to the corresponding calibrated logarithmic curve [25]. PerioPaper was stored in a 50 µL mixture of phosphate-buffered saline (PBS) and protease inhibitors (cOmplete™, Sigma-Aldrich, St. Louis, MO, USA) in plastic sealable Eppendorf tubes and frozen at -80 °C until analysis.

### Enzyme-linked immunosorbent assay

The solution collected using PerioPaper was vortexed for 10 min to elute and then centrifuged at 300 rpm for 10 min at 4 °C. Subsequently, centrifugation was performed at 12,000 rpm for 2 min. The resulting supernatant was collected, and five supernatants were combined to yield a total volume of 250 µL. ET-1 levels were measured using the Quantikine® Enzyme-linked immunosorbent assay (ELISA) Endothelin-1 Immunoassay kit (R&D Systems, MN, USA), while IL-1β levels were measured using the Quantikine® ELISA Huma IL-1β/IL-1F2 kit (R&D Systems). ELISA procedures were performed according to the manufacturer’s instructions. Sites with cytokine concentrations below the detection limit of the assay were recorded as 0. These biomarker concentrations were adjusted for the amount of PISF and expressed as ET-1 (ρg/site) and IL-1β (µg/site) [26].

### Statistical analysis

G*Power 3.1.9.6 software was utilized for sample size calculations, with an effect size of 0.8, a statistical power of 80%, and a significance level of 95% (α < 0.05), two-tailed. Based on these parameters, a minimum of 21 implants were required for each group to detect a difference between the groups, which served as the sample size requirement of the study. Statistical analyses were performed using Bell Curve for Excel (Social Survey Research Information Co., Ltd., Tokyo, Japan). Data normality was evaluated using the Shapiro–Wilk test. The Kruskal–Wallis test was employed to determine statistically significant differences between groups for each independent variable, with subsequent Steel–Dwass adjustment. The diagnostic accuracy of the biomarker candidates for distinguishing peri-implantitis or peri-implant mucositis from healthy implants was evaluated using receiver operating characteristic (ROC) curve analysis and the area under the ROC curve (AUC). Each biomarker, along with its adjusted logistic regression model (adjusted for sex and age of the dental implant), was adjusted. The Youden index was utilized to determine the optimal cut-offs from the ROC curves for each biomarker (unadjusted and adjusted models). Diagnostic sensitivity and specificity were calculated for each biomarker using a cut-off value to assess classification quality. Statistical significance was set at *p* < 0.05.

## Results

### Research participants

Age, sex, implant placement site, and clinical and radiographic evaluations of the patients are shown in Table 1. Among the 76 implants, 29 were classified as healthy (38.2%), 22 showed signs of mucositis (28.9%), and 25 displayed signs of peri-implantitis (32.9%). No significant bias was observed among the three groups in terms of demographic variables or sampling sites. Regarding periodontal parameters, the PPD, mPI, and mGI scores were increased in the mucositis and peri-implantitis groups compared to those in the healthy group.

**Table 1:** Peri-implant health, peri-implant mucositis, and peri-implantitis patient characteristics

Variable		Peri-implant condition		
		Healthy (*n* = 29)	Mucositis (*n* = 22)	Peri-implantitis (*n* = 25)
BOP (+/-)		(0/29)	(22/0)	(25/0)
ABL (mm)	^b^	0.03 (0-0.07)	0.05 (0.02–0.12)	0.42 (0.33–0.61)
Demographic variables				
Age	^a^	70.8 (7.5)	74.9 (8.8)	68.9 (8.4)
	^b^	69 (54–92)	72 (64–92)	69 (54–83)
Gender	Female	12	12	11
	Male	17	10	14
Sampling site				
Jaw	Maxilla	12	7	7
	Mandible	17	15	18
Location in arch	Incisor	9	5	10
	Premolar	10	8	4
	Molar	10	9	11
Implant system	BL	27	20	23
	TL	2	2	2
Peri-implant parameters				
PPD (mm)	^b^	3 (2–4)	4 (3–5)	5 (2–8)
mPI	^b^	0 (0–3)	2 (2–3)	2 (2–3)
mGI	^b^	0 (0–1)	2 (2–3)	2 (2–3)

*Abbreviations* BOP, Bleeding on probing; ABL, Average annual bone loss; BL, Bone level implant; TL, Tissue level implant; PPD, Pocket probing depth

^a^Data presented as mean (SD), ^b^Data presented as median (IQR)

### Comparison of PISF volumes

**Fig. 2:**
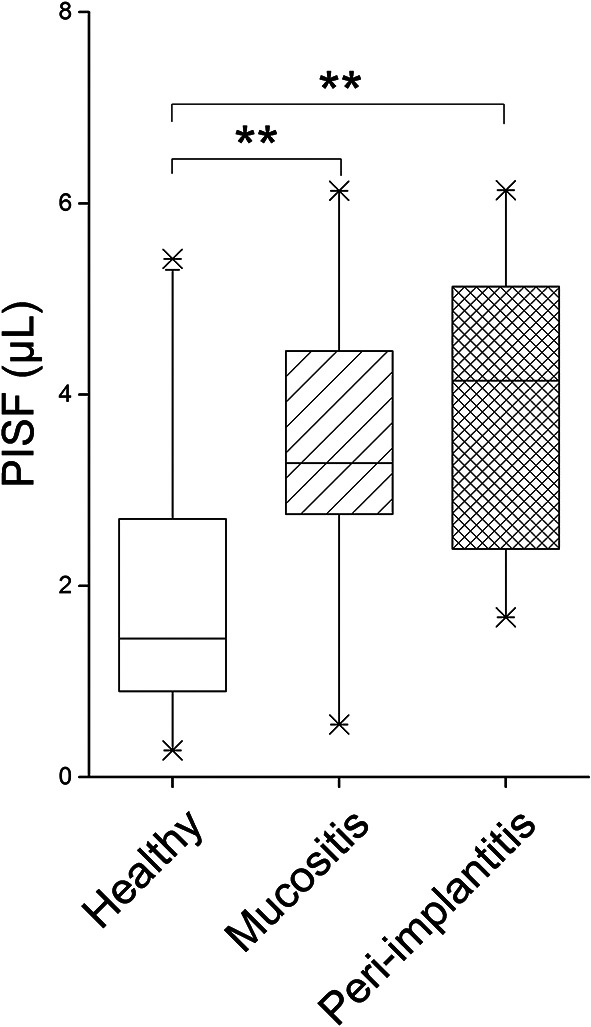
PISF volume in the healthy, mucositis, and peri-implantitis groups The median PISF volume was 1.45 µL in the healthy group, 3.28 µL in the mucositis group, and 4.15 µL in the peri-implantitis group. (*: *p* < 0.05, ** : *p* < 0.01, Kruskal–Wallis test, Steel–Dwass test)

The median PISF volume for each group was as follows: 1.45 (0.89–2.70) µL for the healthy group, 3.28 (2.76–4.45) µL for the mucositis group, and 4.15 (2.39–5.13) µL for the peri-implantitis group. Notably, the PISF volume in the healthy group was significantly lower than that in the mucositis and peri-implantitis groups (*p* < 0.01). However, no significant difference was observed in the PISF volumes between the mucositis and peri-implantitis groups (Fig. 2).

### Comparison of biomarkers

**Fig. 3:**
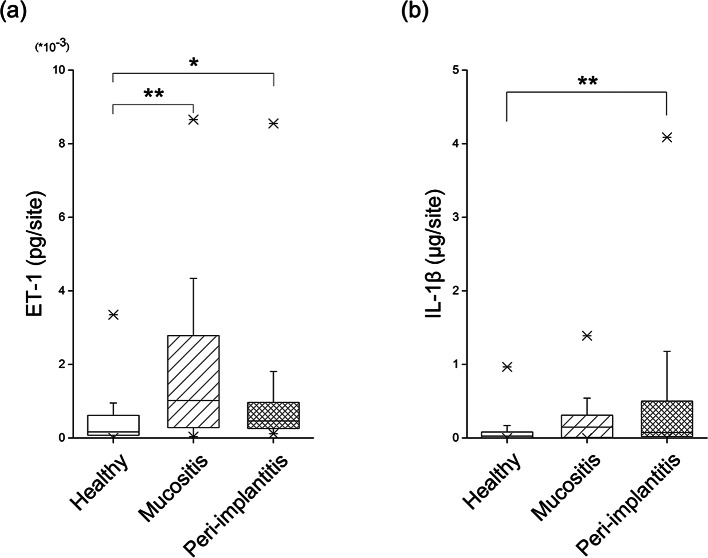
Biomarker expression in the healthy, mucositis, and peri-implantitis groups (**a**) The median values for ET-1 concentration were 0.17 × 10^− 3^ pg/site in the healthy group, 1.02 × 10^− 3^ pg/site in the mucositis group, and 0.47 × 10^− 3^ pg/site in the peri-implantitis group. Between-group assessments showed significantly higher ET-1 expression in the mucositis (*p* < 0.01) and peri-implantitis (*p* < 0.05) groups compared to that in the healthy group (**b**) The median values of IL-1β concentration were 0.03 µg/site in the healthy group, 0.15 µg/site in the mucositis group and 0.08 µg/site in the peri-implantitis group. The IL-1β levels in the peri-implantitis group were significantly higher than those in the healthy group (*p* < 0.01). (*: *p* < 0.05, **: *p* < 0.01, Kruskal–Wallis test, Steel–Dwass test)

The median values for ET-1 in each group were as follows: 0.17 × 10^− 3^ (0.07 × 10^− 3^ – 0.61 × 10^− 3^) ρg/site in the healthy group, 1.02 × 10^− 3^ (0.34 × 10^− 3^ – 2.75 × 10^− 3^) ρg/site in the mucositis group, and 0.47 × 10^− 3^ (0.26 × 10^− 3^ – 0.97 × 10^− 3^) ρg/site in the peri-implantitis group. Between-group assessments revealed significantly higher ET-1 levels in the mucositis (*p* < 0.01) and peri-implantitis (*p* < 0.05) groups than those in the healthy group. The median values for IL-1β in each group were as follows: 0.03 (0.01–0.08) µg/site in the healthy group, 0.15 (0.09–0.30) µg/site in the mucositis group, and 0.08 (0.02–0.51) µg/site in the peri-implantitis group. IL-1β levels in the peri-implantitis group were significantly higher than those in the healthy group (*p* < 0.01) (Fig. 3).

### Comparative evaluation of the diagnostic potential of each biomarker using ROC curves

**Fig. 4:**
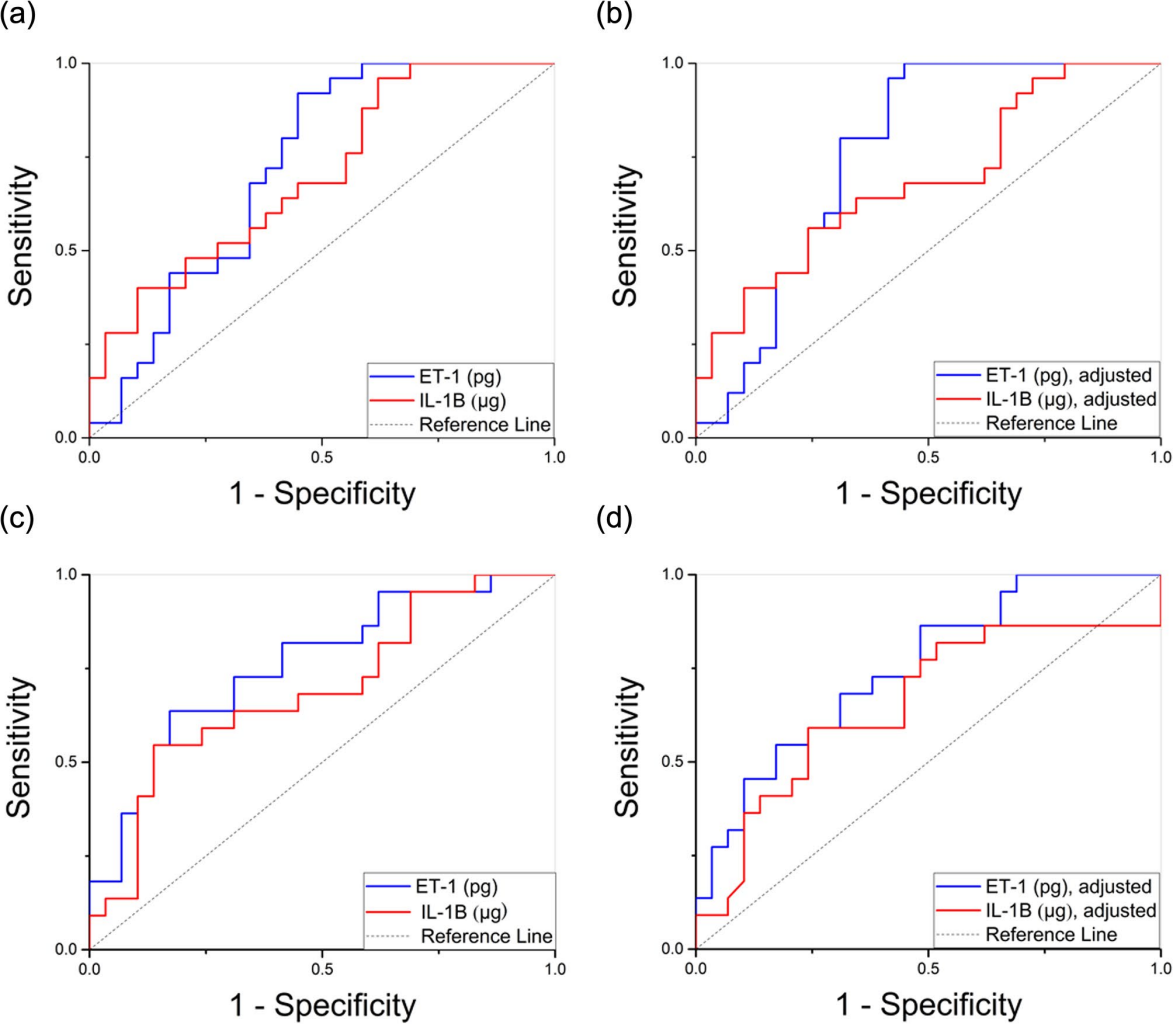
(a and b) Receiver operating characteristic (ROC) analysis showing the diagnostic ability of ET-1 and IL-1β to differentiate healthy groups and peri-implantitis: (**a**) univariable (*n* = 54), (**b**) adjusted for gender, age (*n* = 54), (c and d) ROC analysis showing the diagnostic ability of ET-1 and IL-1β to differentiate individuals in the healthy and peri-implant mucositis groups: (**c**) univariable (*n* = 51), (**d**) adjusted for gender, age (*n* = 51)

The ROC curves were used to evaluate the diagnostic ability of ET-1 and IL-1β for peri-implantitis and peri-implant mucositis, respectively. The ROC curves for ET-1 and IL-1β were generated using univariable analysis and two models adjusted for age and sex (Table 2; Fig. 4).

Graphs depicting the peri-implantitis group as positive and the healthy group as negative are shown in Fig. 4 (a) and (b), respectively. For peri-implantitis, the AUC for ET-1 was 0.72 (95% CI = 0.58–0.86, *p* < 0.01), with a cut-off value of 0.21, yielding a sensitivity of 92% and a specificity of 56% in the univariable analysis. In the adjusted analysis, the AUC for ET-1 was 0.76 (95% CI = 0.63–0.89, *p* < 0.01), with a sensitivity of 80% and specificity of 79% at a cut-off value of 0.41. The AUC for IL-1β was 0.70 (95% CI: 0.56–0.84, *p* < 0.01) in the univariable analysis, with a cut-off value of 0.44, sensitivity of 64% and specificity of 59%. In the adjusted analysis, the AUC for IL-1β was 0.69 (95% CI: 0.54–0.83) with a sensitivity of 64% and specificity of 66% at a cut-off value of 0.39.

**Table 2:** Diagnostic performance comparison using ROC curves

**(a) Comparison of healthy implants and peri-implantitis**							
	Biomarker	Cut-off value	AUC value	95% Cl	*p*-value	Sensitivity	Specificity
Univariable model	ET-1	0.21	0.72	0.58–0.86	< 0.01	92	56
	IL-1β	0.44	0.70	0.56–0.84	<0.01	64	59
Adjusted Model^†^	ET-1	0.41	0.76	0.63–0.89	< 0.01	80	79
	IL-1β	0.39	0.69	0.54–0.83	0.01	64	66
**(b) Comparison of healthy implants and peri-implant mucositis**							
	Biomarker	Cut-off value	AUC value	95% Cl	*p*-value	Sensitivity	Specificity
Univariable model	ET-1	0.89	0.76	0.63–0.89	< 0.01	63	83
	IL-1β	0.15	0.69	0.54–0.84	0.01	59	76
Adjusted Model^†^	ET-1	0.39	0.75	0.61–0.88	< 0.01	68	69
	IL-1β	0.46	0.65	0.49–0.81	0.06	59	76

*Abbreviations*^†^Adjusted for age and gender ; ET-1, Endothelin-1 ; IL-1β, Interleukin 1β ; 95% Cl, 95% confidence interval

Graphs illustrating the peri-implant mucositis group as positive and the healthy group as negative are shown in Fig. 4 (c) and (d). For peri-implant mucositis, the AUC for ET-1 was 0.76 (95% CI: 0.63–0.89, *p* < 0.01), with a sensitivity of 63% and specificity of 83% at a cut-off value of 0.89 in the univariable analysis. In the adjusted analysis, the AUC for ET-1 was 0.75 (95% CI: 0.61–0.88, *p* < 0.01) with a sensitivity of 68% and specificity of 69% at a cut-off value of 0.39. The AUC for IL-1β was 0.69 (95% CI: 0.54–0.84, *p* < 0.01) in the univariable analysis with a sensitivity of 59% and specificity of 76% at a cut-off value of 0.15. In the adjusted analysis, the AUC for IL-1β was 0.65 (95% CI: 0.49–0.81) with a sensitivity of 59% and specificity of 76% at a cut-off of 0.46

## Discussion

This study revealed a significant increase in ET-1 levels in the PISF of patients diagnosed with peri-implantitis compared to those with healthy peri-implant conditions. Interestingly, patients with peri-implant mucositis also showed a significant increase in ET-1 levels, which differs from previously reported [13] biomarkers that were found to be elevated only in cases of peri-implantitis.

Consistent with the findings of previous research [27], peri-implant disease samples exhibited elevated PISF compared to that of healthy peri-implant regions. While ET-1 is highly expressed in keratinocytes, it may also be expressed in tissues in peri-implant mucositis (an early stage of peri-implant diseases) due to increased capillary permeability resulting from inflammation, facilitating faster passage through the vessel wall than normal, leading to increased PISF levels. IL-1β, a cytokine with a large molecular weight of approximately 17.5 kDa. may exhibit poorer membrane permeability compared to that of peptides. Therefore, IL-1β levels may be elevated in peri-implantitis, where tissue destruction around the implant allows for easier passage of the protein through vessel walls [16]. While most studies on peri-implant diseases focus on cytokines, this study suggests that utilizing peptides with small molecular weights as biomarkers could potentially enable earlier diagnosis. However, it is important to acknowledge that peptides with low molecular weights may be difficult to detect using conventional ELISA methods. Therefore, an ELISA with a high degree of detection sensitivity is necessary to measure minute amounts of peptides effectively for accurate diagnosis and evaluation.

ET-1 plays an important role in the pathogenesis of periodontitis; previously reported [19, 20] found that exposure to *Porphyromonas gingivalis* induces ET-1 secretion from endothelial cells and stimulates inflammatory cytokines such as IL-1β and TNF-α. ET-1 and inflammatory cytokines are believed to be interdependent and establish an inflammatory loop that promotes osteoclastogenesis and causes alveolar bone resorption [21, 28, 29]. While periodontitis and peri-implantitis have distinct supporting tissue structures, they share many clinical features and biomarkers [30]. Consequently, it is presumed that a comparable mechanism operates in the peri-implant environment.

The elevated levels of ET-1 in peri-implant mucositis and decreased levels in peri-implantitis may be explained by the balance between pro- and anti-inflammatory effects through regulatory feedback. In particular, ET-1 is a pro-inflammatory peptide and is therefore upregulated in the early and acute phases of inflammation. In chronic inflammation, such as peri-implantitis, prolonged exposure may lead to the downregulation of ET-1 due to cytokine and cellular changes.

ROC curve analysis was performed to determine whether discrimination of the presence and severity of inflammation in the peri-implant mucosa was feasible. The AUC value for ET-1 was 0.76 with 80% sensitivity and 79% specificity. These results suggest that ET-1 is more effective than IL-1β in distinguishing peri-implantitis from healthy peri-implant conditions. Additionally, ROC analysis comparing healthy peri-implants to those with peri-implant mucositis yielded an AUC of 0.75 with a sensitivity of 68% and a specificity of 69%. The diagnostic accuracy of peri-implant mucositis was comparable to that of peri-implantitis. Given the significant increase in ET-1 levels observed in peri-implant mucositis, ET-1 appears to be more effective than IL-1β in the screening and early diagnosis of peri-implant diseases.

Diagnosing peri-implant mucositis is challenging because of the lack of clinical symptoms and the complex interplay of various factors. Therefore, unlike peri-implantitis, diagnosing peri-implant mucositis at a reversible and treatable stage is beneficial. Even if there are no clinical symptoms, an increase in the frequency of maintenance and localized prophylactic treatment may prevent future inflammation and bone resorption if ET-1 is elevated.

This study had certain limitations. For example, the study design followed that of a previous study [31]. In this design, implants within a single patient may be assigned to different groups. Patient-related factors were analyzed with implant as the unit, and thus the interdependence of implants within the same patient was not considered. Therefore, it may not be possible to infer causal relationships between risk factors.

However, the marked differences in ET-1 between implants in this study could be attributed to different physiological responses at inflammatory and healthy sites. Since clear differences in implant behavior between the different groups were also observed, statistical variability may not necessarily increase significantly, and grouping was judged to be reasonable. Another limitation is that biomarkers may not always be present during PISF collection due to systemic or local factors. Further research and longitudinal studies are warranted to better comprehend the relationship between ET-1 and peri-implant diseases and to integrate ET-1 as a biomarker into treatment protocols.

Future studies investigating the role of ET-1 in peri-implant diseases hold promise for developing novel treatment approaches for peri-implant diseases. Son et al. [32] demonstrated that an endothelin receptor antagonist, bosentan, partially ameliorates alveolar bone resorption and immune cell infiltration, suggesting a potential new avenue for treating periodontitis. As there are currently no established pharmacological treatments for peri-implant diseases, repurposing bosentan, which has already been approved for the treatment of pulmonary hypertension, may be a promising therapeutic for peri-implantitis by mitigating inflammation.

## Conclusion

Our results suggest that the presence of ET-1 in PISF may play a role in peri-implant diseases. Additionally, the significant increase in ET-1 levels observed in peri-implant mucositis indicates that combining ET-1 with conventional examination methods may improve the accuracy of inflammation evaluation.

## Data Availability

No datasets were generated or analysed during the current study.

## Abbreviations

ET-1: Endothelin-1
PPD: Probing Pocket Depth
BOP: Bleeding on probing
PISF: Peri-implantitis Sulcus Fluid
IL-1β: Interleukin-1β
TNF-α: Tumour Necrosis Factor alpha
MMP-8: Matrix Metalloproteinase 8
mPI: modified Plaque Index
mGI: modified Gingival Index
MBL: Marginal Bone Loss
ROC: Receiver Operating Characteristic
AUC: Area Under the Curve
